# Long Branch Effects Distort Maximum Likelihood Phylogenies in Simulations Despite Selection of the Correct Model

**DOI:** 10.1371/journal.pone.0036593

**Published:** 2012-05-09

**Authors:** Patrick Kück, Christoph Mayer, Johann-Wolfgang Wägele, Bernhard Misof

**Affiliations:** Zoologisches Forschungsmuseum Alexander Koenig (ZFMK), Bonn, Germany; East Carolina University, United States of America

## Abstract

The aim of our study was to test the robustness and efficiency of maximum likelihood with respect to different long branch effects on multiple-taxon trees. We simulated data of different alignment lengths under two different 11-taxon trees and a broad range of different branch length conditions. The data were analyzed with the true model parameters as well as with estimated and incorrect assumptions about among-site rate variation. If length differences between connected branches strongly increase, tree inference with the correct likelihood model assumptions can fail. We found that incorporating invariant sites together with 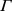 distributed site rates in the tree reconstruction (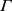
+I) increases the robustness of maximum likelihood in comparison with models using only 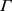. The results show that for some topologies and branch lengths the reconstruction success of maximum likelihood under the correct model is still low for alignments with a length of 100,000 base positions. Altogether, the high confidence that is put in maximum likelihood trees is not always justified under certain tree shapes even if alignment lengths reach 100,000 base positions.

## Introduction

Maximum likelihood (ML) tree inference has been shown to be statistically consistent for binary trees with finite branch lengths under correct model and model parameter assumptions as sequence length increases to infinity [1]–[5]. Thus, ML tree inference will converge on the true tree as more and more data are accumulated [5], [6]. Additionally, ML is said to be robust against model violations [2], [6]–[11] and thus, even oversimplified likelihood models are said to find the correct tree in most instances if branch lengths are well balanced [12].

The ML method is certainly more robust and more efficient than other methods [2], [6]–[9], [13]–[21]. This has led to a widespread application and acceptance of ML tree inference. Since its introduction into phylogenetics, the degree of ML robustness and efficiency has been assessed using 4-taxon tree simulations. Setups in which ML methods can potentially fail or become inefficient on trees with more than four taxa have not been intensively studied in e.g. Fukami & Tatento [22], Kuhner [23], Huelsenbeck [24], and Pol & Siddal [25]). Since phenomena like taxon-slippage in larger trees due to signal erosion (class II long branch effects sensu Wägele & Mayer [26]) cannot be seen in four-taxon trees, we address the robustness and efficiency of ML methods to different long branch effects in an 11-taxon setup. We show that ML methods indeed reconstruct correct topologies in a wide parameter range, but we also discovered instances in which ML methods reconstruct the wrong tree for relatively long alignments even under correct model assumptions. These effects, which have not been studied previously, are potentially common in empirical data.

It is well known that if among-site rate variation (ASRV) is ignored in tree reconstruction, the ML approach underestimates substitution rates, and these estimates become progressively worse with increasing evolutionary distances [27]. Ignoring ASRV makes ML tree inference susceptible to long branch attraction [4], [6], [7], [11], [13], [15], [16], [19], [28]–[30]. Therefore, ASRV is, apart from other important advances like the consideration of multiple substitutions or basing phylogenetic inference on a sound statistical footing, another powerful improvement brought to model-based ML reconstruction methods. Three possibilities to account for rate variation are the “invariant sites model (I)”, the “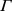 distributed rates model” (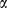 shape parameter) and a combination of both models (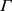
+I). The invariant sites parameter assumes an estimated fraction of sites to be invariable while remaining sites are assumed to evolve at an equal rate. Under the 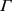-model, substitution rate heterogeneity among sites is modelled using a 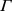 distribution. A bell-shaped 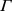 distribution caused by an 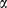 value greater than 1 implies a more or less constant substitution rate among sites whereas a reverse-J shaped 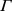 distribution caused by 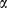 values lower than 1 means a stronger rate variation [15]. The lower the 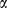 value, the higher the rate heterogeneity among sites.

Early studies argue that combining both models (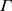
+I) into a mixed-distribution model should lead to a significant improvement of the heterogeneity estimation in comparison to invariable sites- or 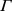-model estimates alone [9], [16], [31]–[33]. However, recently published studies relied on the exclusive application of the restricted 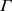-model (e.g. [34]–[38]). One argument is that parameters of the 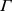
- and invariant sites model cannot be optimized independently. This can lead to problems during model parameter optimization due to multiple optima in the likelihood function [39], [40]. The shape parameter of the 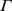 distribution and the invariant sites estimation are indeed strongly correlated and subject to large sample variance [31], [32], [41]. The correlation makes it difficult to distinguish between truly invariable and slowly evolving sites, especially in the case of alignments with a small number of sequences. However, if many taxa are included (N>20), it is said that the mixed-distribution model can be reliably estimated [32], [41]. Erroneous estimates of one parameter can be compensated by the other. Erroneous estimates of both together can fit the data such that the likelihood score changes only marginally [32]. The recent tendency in the literature to prefer the application of the ASRV 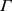 alone mirrors the uncertainty in the modelling of ASRV. We have addressed the important question whether 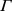
+I models are superior over pure 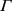 models and whether the parameters could be estimated correctly for a taxon set of just 11 taxa. Furthermore, we investigated how deviations from the simulated 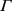 parameter affects the reconstruction success.

**Figure 1:**
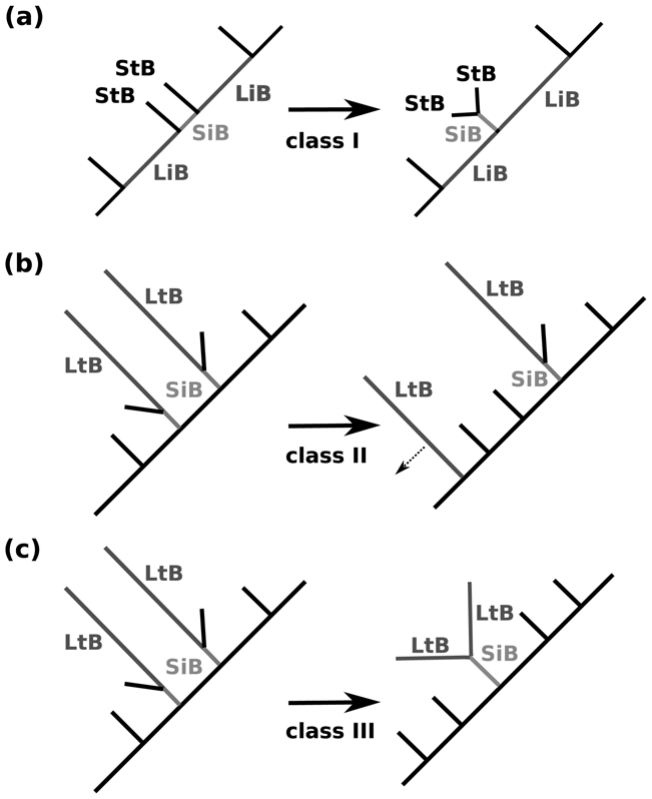
Long branch effects. (a) class I effect (attraction due to symplesiomorphies): two short terminal branches (StB), separated by a short internal branch (SiB) are grouped together due to true homologies. The true homologies are mainly produced by plesiomorphies which can only be identified in rooted topologies. The rest of the tree is found at the ends of two long internal branches (LiB) on either side of the two short branches. (b) class II effect: At least one of the two long teminal branches (LtB) slides down the tree or appears elsewhere in the resulting tree topology, mainly due to signal erosion along the corresponding long terminal branch (c) class III effect: Two long terminal branches (LtB) separated by more than one internal branch are attracted in direct analogy to the “Felsenstein” case, which is due to dominance of change similarities over homologies. The two different tree shapes of the true topologies were transferred onto the two model topologies which were used in our data simulations (Figure 2).

No model can be assumed to be entirely correct for real data [19]. Long branch artefacts (LBA) are therefore not only theoretical concepts, but also real phenomena [24], [26], [42]. The “classical long branch case” (Figure 1a) which is caused by the misleading effect of parallel substitutions on long branches [2] is well studied and affects mainly the maximum parsimony method. In a topology of more than four taxa, (i) the case when two internal long branches are separated by a short internal branch in a rooted tree with more than four taxa (Figure 1a), may lead to misplacement of the two terminal taxa adjacent to the short inner branch. We call this phenomenon the class I effect (following Wägele & Mayer [26]). This effect is mainly produced by plesiomorphies. Note that these can only be identified in rooted tree topologies and that they are true homologies, in contrast to the chance similarities typical for the Felsenstein Zone. (ii) The case when a single long branch slips down the tree towards the outgroup or appears elsewhere, mainly due to signal erosion (Figure 1b), has been coined the class II effect. (iii) Finally, the case described in detail by Felsenstein [2], namely the attraction of long terminal branches due to the dominance of chance similarities over homologies, is named the class III effect. Note that it is relevant to find out if long terminal branches are also attracted due to class III effects when they are separated by more than one internal branch. This can only be tested in multiple taxon tree topologies (Figure 1c).

## Results

### Reconstruction Success of Topology A

**Figure 2:**
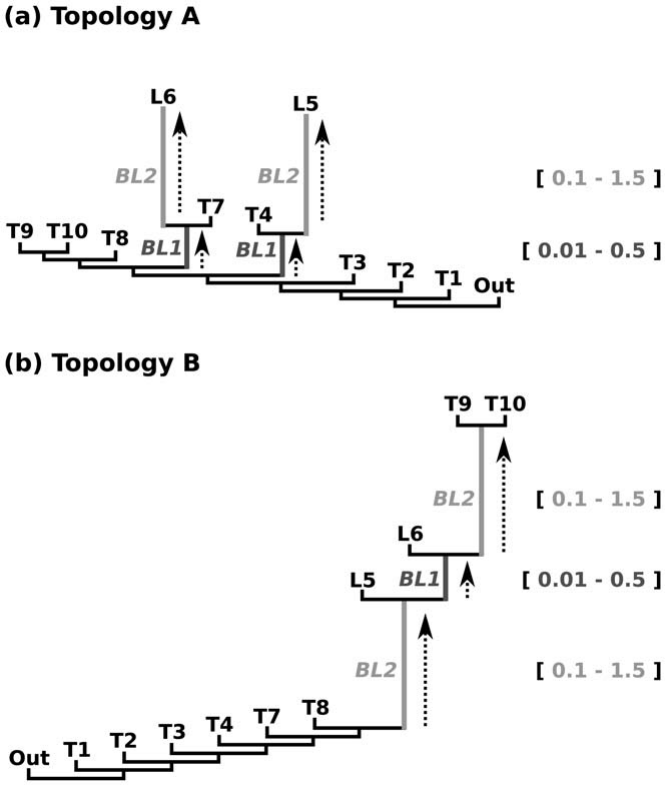
Two sets of simulations. Given model topology for a) Topology A: stepwise elongation of two terminal branches (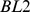) under different ancestral branch lengths (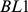) and b) Topology B: stepwise elongation of two internal branches (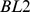) under different lengths of an intermediate branch (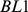). Topology A was used to identify class II and III effects (following tree shape of Figure 1b and Figure 1c), Topology B was used to identify class I effects (following tree shape of Figure 1a).

**Figure 3:**
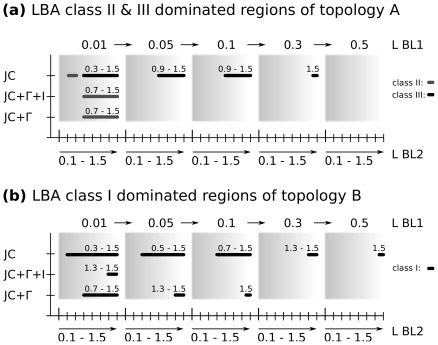
Selected results of ML reconstuctions for 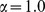 under the mixed-distribution model (JC+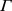
+I) and the 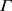 distributed model (Jukes-Cantor+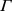). Class III (“Felsenstein effect”), Class I (attraction due to symplesiomorphies), and Class II (random error probably due to signal erosion) inferred from 100 simulation repeats for each branch length combination and alignment length. Each individual plot corresponds to a fixed branch increase of 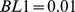 (Figure 2) and fixed reconstruction scheme with the models JC+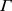 (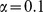) or JC+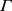
+I (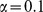; 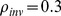). Branch length differences increase from left to right by increasing branch 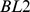 in discrete elongation steps (0.1–1.5). Four successive data points (belonging to one cell in the plot) correspond to four alignment lengths (2,000, 3,000, 4,000, 10,000 base pairs). Alignment corresponding branch lengths of 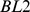 are shown above each subfigure. The y-axis depicts the reconstruction success of the 100 simulation repeats (N) for a) Topology A (Figure 2a) and b) Topology B (Figure 2b).

**Figure 4:**
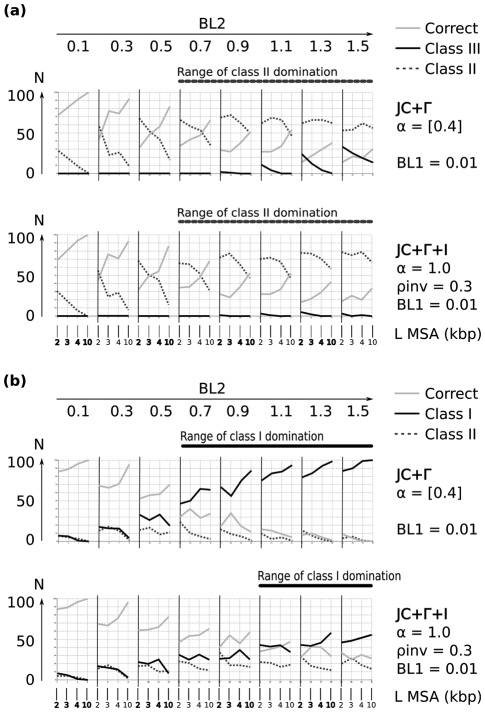
Occurence of long branch artefacts (LBA). Ranges of branch length differences between 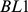 and 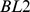 (see Figure 2) in which LBA dominated tree reconstruction with investigated model assumptions in the majority of repeat steps, summarized over all alignment lengths. Dominated ranges of long branch artefacts are shown by bar charts. Single bars correspond to fixed ranges of lengths for 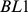 and 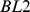 in which lengths of 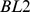 increase from 0.1–1.5 within each box (x-axis; lower scale). Length of 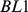 increases with each box from 0.01–0.5 (x-axis; upper scale). a) Domination of class II and class III effects are found in topology A (Figure 2a). b) Domination of the class I effect is found in topology B (Figure 2b). Corresponding branch lengths of 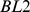 are also shown above each bar plot. Note, that ML delivered identic tree reconstruction success for estimated and correct model assumptions of JC+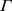
+I.

Topology A (Figure 2a) was designed to test for class II (signal erosion) and class III effects (attraction due to chance similarities). If the true proportions of invariant sites (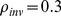) and ASRV (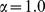) are given or estimated for datasets of Topology A (Figure 2a) by using a mixed-distribution model of ASRV (JC+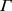
+I) or if estimated by a 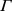 distribution model alone (JC+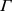), ML is able to infer predominantly correct trees under most of the internal branch lengths (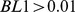) even if terminal branch lengths are extremely long (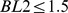) (Figure 3a and Figure S1). Class II effects, where one single long branch slips down the tree towards the outgroup or appears elsewhere, predominate only in the majority of simulations if short internal branch lengths 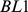 are very low (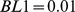). This implies weak signal supporting internal nodes. Under these conditions class II effects are found even under moderate lengths of long terminal branches (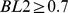) (Figure 3a and Figure 4a). Long branch attraction of both terminal branches (class III effects) were only rarely seen if terminal branches are distinctly long (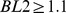) and alignment lengths short (2000 bp), but appear more often if JC is used for tree inference with a 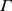 distribution model alone (Figure 4a). As expected, ML performs worse if rate heterogeneity is not considered at all (Figure 3a and S1). In this case, especially long branch effects due to attraction of long terminal branches (class III) are present in the majority of simulations except when internal branch lengths get very large (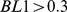, implying better support for inner nodes). The range in which class III effects predominate in tree reconstructions without consideration of rate heterogeneity decreases continuously with increased branch lengths of the short internal branches 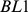 (Figure 3a and Figure S1).

**Figure 5:**
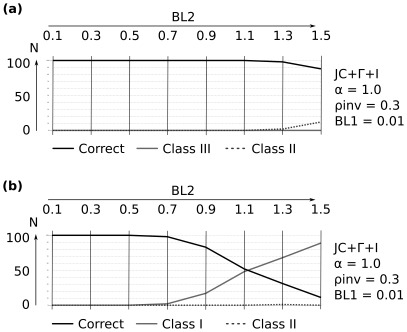
Reconstruction success of ML (100,000 base positions). a) Topology A (Figure 2a) and b) Topology B (Figure 2b) under alignment lengths of 100,000 base positions if model assumptions are identical to the simulated parameters (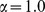; 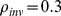). Branch length differences increase from left to right by increasing 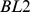 in discrete steps (0.1–1.5) while 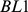 is kept constant (0.01). The y-axis depicts the reconstruction success of the 100 simulation repeats (N).

While the class II effect (signal erosion) predominates tree inference even under correct model assumptions (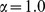; 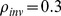) and moderate sequence lengths of 10,000 bp when 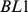 is very small (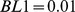), ML correctly resolves nearly all trees under these conditions when sequence lengths are extended to 100,000 bp under equal ML parameter settings (Figure 5a). In general, the performance of ML inference in our simulations is mostly afflicted by large branch length differences, less so by wrong model assumptions.

### Reconstruction Success of Topology B

Topology B was designed to test for class I effects (symplesiomorphy effect). The major difference to topology A is that the evolving sequence passes through two long branches, while in topology A the two long branches are parallel. Even if the correct proportion of invariant sites (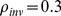) and ASRV (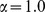) are assumed, ML is not able to infer correct trees of topology B (Figure 2b) in the majority of simulations if the length of the short internal branch (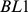) is small (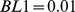) and the lengths of the two long internal branches (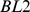) are large (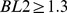) (Figure 3b and Figure 4b). When the mixed-distribution model (JC+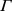
+I) is used, class I effects (symplesiomorphy effects) start to predominate in the majority of tree reconstructions of topology B if the lengths of the long internal branches (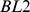) is large (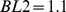) except for the alignment length of 10,000 bp. If 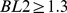, class I effects are also found for alignment lengths of 10,000 bp (Figure 3b and Figure 4b). The frequency of class I effects is even higher if data stets are analysed with JC and the 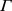 distribution model alone (Figure 3b and Figure 4b). If the short internal branch length (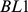) is small (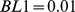), class I effects already predominate with JC+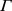 in the majority of repeat steps if both long internal branches (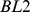) 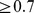. In contrast to JC+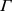
+I, predomination of class I effects is additionally found if lengths of the short internal branch (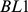) are larger than 0.01 (Figure 3b and Figure S1). If ML is used without consideration of ASRV, tree reconstruction success for topology B is worse than described for topology A (Figure 3 and Figure S1). In contrast to topology A, the high frequency of wrong trees does not disappear in topology B under correct model assumptions when sequence alignment lengths rise to 100,000 bp (Figure 5b). This is in agreement with the fact that the symplesiomorphy 6effect is a systematic error, not only caused by random variations but inforced by shared homologies. It can be overcome with a better taxon sampling [26].

### Maximum Likelihood Values

Likelihood values of single trees become higher if among-site rate variation is considered. All trees affected by long branch artifacts show likelihood scores that are nearly identical to those of correctly resolved topologies of corresponding sequence lengths and parameter assumptions. Likelihood values of all reconstructed trees corresponding to the results of Figure 3 are shown in Figure S2. It is important for work with empirical data that distinct differences in likelihood scores between wrong and correct topologies could not be observed in many cases even if the ML parameters used for inference were nearly identical to their true values.

### Parameter Estimates of 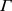 and I

If 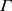 was estimated alone (JC+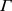), 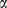 was estimated on average to 0.4 under small branch length differences of 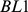 and 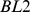. If length differences between 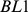 and 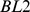 got larger, the estimated 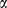 value decreased continiously with increasing branch length differences until 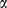 was estimated on average to 3.5 (Figure S3). If 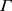
+I were estimated together, 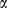 was estimated on average slightly higher as simulated for larger branch length differences (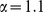). The proportion of invariant sites was on average consistently estimated to 0.3 independent of corresponding branch length conditions. The tree reconstruction success of both (estimated and correct values of JC+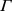
+I) settings was found to be nearly identical (Figure S1). All parameter estimates are presented as Figure S3.

## Discussion

For alignment lengths in the range of 2,000–10,000, the reconstruction success was investigated for i) correct as well as estimated model parameters (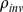 and 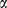) with a mixed-distribution model (JC+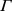
+I), ii) a 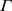 distribution model in which 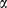 was estimated alone (JC+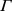), and iii) without considering rate heterogeneity (JC).

As expected, our results show that incorporating rate heterogeneity leads to an increased reconstruction success of ML (provided that the data includes rate heterogeneity). This has also been observed in previous studies, e.g. [9], [12], [24], [32], [33], and is not surprising.

The inclusion of a mixed-distribution model (JC+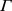
+I) improves tree estimation over analyses using a 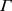 distribution model alone. Especially in case of topology B, JC+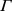
+I recovered the correct topologies under a wider range of branch lengths as JC+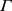 (Figure 3b and Figure 4b). This supports the results of Sullivan et al. [32] as well as Anderson & Swofford [9] who showed that ML recovers topologies best if a 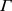
+I model is used and contradicts the assumptions that exclusive application of the restricted 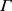-model is sufficient, e.g. [34]–[38]. Whether the higher tree reconstruction success of the mixed-distribution model associated with topology B will also be true with empirical data has to be tested in further studies. For a combination of very short 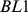 and long 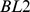, ML performs poorly, even if a mixed-distribution model is used in the tree reconstruction (Figure 3 and Figure 4). The lower reconstruction success for the very short length of 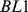 (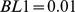) cannot be due to random choice of a most-likely topology when there is no phylogenetic signal (star topology). In such cases ML is expected to “choose” at random from the set of all plausible topologies [6], [8]. This would be the expected behavior of ML when information of ancestral states is completely lacking. However, this is neither the case for topology A (Figure 4a) nor for topology B (Figure 4b). Despite large length differences between ancestral (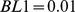) and terminal branches (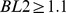) for Topology A, ML was still able to infer correct topologies more often than can be attributed to chance. Similarly, the incorrect trees that place taxon L5 and L6 in a sister group relationship due to the class I effect (Figure 1a) appear more often for Topology B than expected by chance. The explanation for this effect is the systematic bias.

ML is not able to recover the true tree for Topology B with large length differences between short (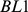) and long branches (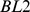), even if the correct model is specified (Figure 5b). This class of topologies has not been investigated before and constitutes a new example for which ML efficiency is low even for long alignments (100,000 bp). With increased sequence length, the class I effect (symplesiomorphy effect) becomes even stronger beyond a certain point of branch length differences of short internal branches (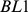) and long internal branches (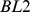) (Figure 4b). However, the proofs of ML consistency mean that there is always some k large enough that having k or more sites will allow the true tree to be inferred with high probability assuming correct model parameters. For the case of 4 taxa, the “inverse Felsenstein zone” is a well known example of reduced ML efficiency where alignment lengths of 100,000 bp are required for an 85% chance to recover the correct topology [6]. It can be expected that our topology and setup yields, what we call, an “inefficient valley of death”, which is similar to the effect found for the “inverse Felsenstein zone” by Swofford et al. [6] where the performance of likelihood declines initially and then improves as sequence length increases. Figure 5b suggests that alignment lengths may need to be in the millions or even higher, meaning the LBA class I problem couldn’t be resolved yet, even in principle for many bacterial genomes. Since we can soon regularly expect data sets of the size of complete genomes, it would be interesting to investigate the extent of this valley, i.e. the necessary alignment length for which ML will reliably find the correct tree. For the topology A which can produce the Felsenstein effect (Figure 2a), ML recovers the true tree efficiently even with large branch length differences of short ancestral branches 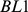 (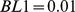) and long terminal branches 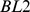 (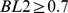) if model assumptions are correct and alignment lengths long (Figure 5a). Our results for this topology are consistent with those found by Swofford et al. [6].

One possible explanation why Topology A and B yield different reconstruction efficiencies could be that the reconstruction of Topology B is in fact more difficult than the reconstruction of Topology A. Because both internal taxa L5 and L6 are separated by a short branch and separated from all other taxa by long branches they will share characters unique for their last common ancestor more often than expected by chance. This will likewise be true for other taxa connected via short branches. Therefore topology B is naturally much harder to reconstruct and given long branch length differences will yield a biased reconstruction error which we see in fact in our simulations.

It is also interesting to note that estimates of 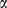 and the invariant sites proportion are very accurately estimated for the 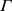
+I models used in the reconstruction. This high accuracy is found for all branch lengths and topologies even in those cases for which the reconstruction success is low (Figure S3). This excludes model misspecification as the source of phylogenetic inaccuracy in analyses in which the tree was inferred using the same parameters as were used to generate the dataset. In those cases, (e.g. Figure 5b) ML consistency implies that the phylogenetic inaccuracy is caused by sampling error. Another possibility is that the heuristic ML searches got stuck in local optima, but this seems rather unlikely for just 11 taxa and JC+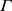
+I. Sullivan et al. [32] argued that the number of taxa is important for the correct estimate of the shape parameter and the number of invariable sites, mainly due to stochastic errors in small samples. The observation that 11 taxa already allow us to find good estimates of the parameters in question could be explained by longer alignments in this study. Further, Sullivan et al. [43] demonstrated on 4-taxon trees that estimates of the 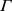 distribution can be strongly influenced by topologies which involve long internal branches. This correlation was not found in our analyses.

As shown in our analyses, the appearance of long branch artefacts, especially of class I effects (symplesiomorphy effect), is not a particular problem of mixed-distribution models. If no invariant sites are estimated in the reconstruction, this model deficiency is partially compensated by a lower estimated value of the 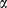 shape parameter (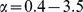), which results in an increased estimate of sites with low and very low substitution rates. Since this compensation is only partial and leads to an overestimation of substitution rates for a certain number of sites, the reconstruction success is lower compared with the application of a 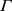
+I model.

Our results show that the risk of obtaining a wrong topology using ML is dependent on the arrangement of the edges (corresponding to which LBA classes the tree is susceptible to). Although our results depend on simulated nucleotid data it can be expected that amino acid sequences are also prone to long branch effects if branch lengths combinations of 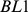 and 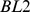 differ strongly from each other, even though the possibility of obtaining long branch effects increase with a decreasing alphabet of character states. It is also clear that good “support values” are no guarantee for the correctness of the tree topology. Also, we have to keep in mind that empirical data can evolve in a much more heterogeneous way than in our simulations. Although we show that ML is not immune to different long branch artefacts, we hope that our work will not be taken as evidence for the continued use of Maximum Parsimony for molecular data. Maximum Parsimony has been shown to be seriously affected by long branch attraction [2], [6], [8], [15]–[17], [19], therefore we consider Maximum Parsimony as entirely inappropriate for molecular data.

## Materials and Methods

### Simulations

We designed two sets of data simulations under different topologies (Figure 2) to detect effects related to long terminal branches (Topology A) and of long internal branches (Topology B). The first set used topology A, which was characterized by a stepwise elongation of two terminal non-neighboring branches (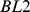). Internal branch lengths (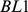) were kept short, but also varied in length (Figure 2a). This setup can potentially produce cases of class II and class III. The second set used topology B, which was characterized by a stepwise elongation of two internal branches (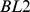) for different lengths of an intermediate internal branch (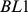) (Figure 2b). This tree topology was used to produce mainly class I effects rather than class II effects. Trees consisted of 11 taxa in which lengths of all remaining branches (*RB*) are kept constant (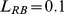). Branch lengths reflect the amount of expected substitution rates per site for corresponding lineages. For each length of 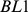 (0.01, 0.05, 0.1, 0.3, 0.5), we increased the length of 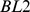 from 0.1 to 1.5 in steps of 0.2. Thus, branch length ratios 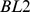/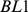 ranged from one-fifth to 150. All alignments were generated with INDELible v.1.01 [44] using the Jukes-Cantor model (JC) of sequence evolution and a mixed-distribution model of 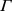
+I for ASRV. All data were simulated with ASRV, shape parameter 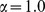, and a proportion of invariant sites 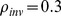. ASRV was modelled using a continuous 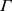-rate distribution while indel events were not simulated. For each branch length-combination of 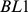 and 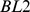, we simulated the evolution of 100 data replicates for each sequence length (2,000, 3,000, 4,000, 10,000 and 100,000 bp). The JC model has been chosen for the simulations (i) since it is better understood than any other model of sequence evolution and (ii) to keep the model parameter space as small as possible. Due to the simple assumptions of the JC-model (each base in the sequence has an equal probability of changing which results in equal frequency of the four bases), the reconstruction success of ML is directly linked to the simulated branch length conditions, sequence lengths, and the ASRV conditions used in each ML analysis.

**Table 1:** The used model parameter settings of ASRV for maximum likelihood analyses.

		Γ		I
JC	+	100		
**JC**	+	**1.0**	+	**0.3**
JC	+	estimate	+	estimate
JC	+	estimate		

Single settings included either 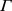 or 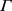
+I parameters (fixed or estimated). Simulated ASRV as well as model parameter setting for additional simulations/analyses of alignment length of 100,000 base positions are highlighted bold.

### Maximum Likelihood Analyses

Trees were inferred with the Jukes-Cantor (JC) model under different parameter settings using PhyML-3.0-linux64 [45], [46] (Table 1). We analyzed the data either (i) with a mixed-distribution model (JC+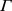
+I) or (ii) with 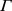 distributed rates, but without estimating the fraction of invariant sites (JC+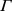). Using the mixed-distribution model (JC+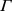
+I), the 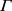 shape parameter 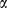 and the fraction of invariant sites were either estimated or set equal to the simulated values (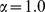 and 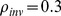). Using the 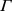 distribution model (JC+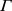), the shape parameter of the 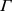 distribution (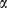) was always estimated from the data. As approximation to non-ASRV (JC), 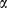 was set to 100 (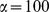). For the alignment length of 100,000 bp, tree reconstruction was only performed under the correct model parameters (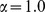 and 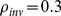). With the discrete gamma model, the number of relative substitution rate categories was set to four (c = 4) and tree topologies and branch lengths were optimized (heuristic search). Maximum likelihood analyses were performed and evaluated with a Perl pipeline, and ran for three months on a Linux Cluster with HP ProLiant DL380 G5 blades (Dual quad core Intel Xeon E5345, 2.33 GHz, 2× 4 MB L2-cache, 1333 MHz Bus, 32 GB RAM).

### Scoring

Wrong topologies were classified into LBA class I, II and III effects (Figure 2). Wrong topologies for which we found a paraphyletic grouping of the two terminal “non-long branches” in topology B were summarized as class I effects. Wrong topologies which showed an attraction of the two long terminal branches in topology A are sampled as class III effects. Wrong topologies for which only one long branch had been misplaced in Topology A and B (probably due to signal erosion) were collectively classified as class II effects. Topologies that did not fit any of these categories like incorrect placements of “background” taxa have not been found in our analyses.

## Supporting Information

Figure S1 ML reconstruction success of simulated paramter and branch length settings.
(TIFF)

Figure S2 ML values of reconstructed topologies.
(TIFF)

Figure S3 ML parameter estimates of reconstructed topologies.
(TIFF)
