# Correction: Long Branch Effects Distort Maximum Likelihood Phylogenies in Simulations Despite Selection of the Correct Model

**DOI:** 10.1371/annotation/0d9fd920-c280-4753-a5a3-e19f372e852b

**Published:** 2012-10-19

**Authors:** Patrick Kück, Christoph Mayer, Johann-Wolfgang Wägele, Bernhard Misof

The legends for Figures 3 and 4 were incorrectly switched. The legend that appears with Figure 3 should be with Figure 4, and the legend that appears with Figure 4 should be Figure 3.

There were also errors in the legend for Figure 4. The correct version of Figure 4 can be seen here: 

**Figure:**
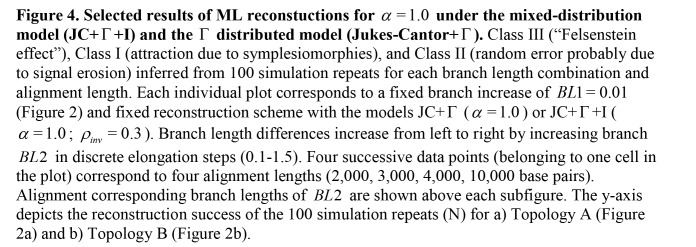


[^] 

